# Direct and Indirect Downstream Pathways That Regulate Repulsive Guidance Effects of FGF3 on Developing Thalamocortical Axons

**DOI:** 10.3390/ijms26157361

**Published:** 2025-07-30

**Authors:** Kejuan Li, Jiyuan Li, Qingyi Chen, Yuting Dong, Hanqi Gao, Fang Liu

**Affiliations:** 1 Department of Cell Biology, School of Basic Medical Sciences, Jiangxi Medical College, Nanchang University, Nanchang 330031, China; 2 Medical Experimental Teaching Center, School of Basic Medical Sciences, Jiangxi Medical College, Nanchang University, Nanchang 330031, China; 3 Queen Mary School, Jiangxi Medical College, Nanchang University, Nanchang 330031, China

**Keywords:** FGF3, thalamocortical axons, thalamus, axon guidance, downstream pathways

## Abstract

The thalamus is an important sensory relay station. It integrates all somatic sensory pathways (excluding olfaction) and transmits information through thalamic relay neurons before projecting to the cerebral cortex via thalamocortical axons (TCAs). Emerging evidence has shown that FGF3, a member of the morphogen family, is an axon guidance molecule that repels TCAs away from the hypothalamus and into the internal capsule so that they subsequently reach different regions of the cortex. However, current studies on FGF-mediated axon guidance predominantly focus on phenomenological observations, with limited exploration of the underlying molecular mechanisms. To address this gap, we investigated both direct and indirect downstream signaling pathways mediating FGF3-dependent chemorepulsion of TCAs at later developmental stages. Firstly, we used pharmacological inhibitors to identify the signaling cascade(s) responsible for FGF3-triggered direct chemorepulsion of TCAs, in vitro and in vivo. Our results demonstrate that the PC-PLC pathway is required for FGF3 to directly stimulate the asymmetrical repellent growth of developing TCAs. Then, we found the FGF3-mediated repulsion can be indirectly induced by *Slit1* because the addition of FGF3 in the culture media induced an increase in *Slit1* expression in the diencephalon. Furthermore, by using downstream inhibitors, we found that the indirect repulsive effect of FGF3 is mediated through the PI3K downstream pathway of FGFR1.

## 1. Introduction

The precise regulation of axonal navigation plays a crucial role in establishing highly ordered efficient neural networks. Numerous classical guidance cues—including Netrins, Slits, Ephrins, Semaphorins, nerve growth factors (NGF), and glial-derived neurotrophic factors (GDNF)—have been identified, each playing specific roles in axon guidance [1,2]. Recently, increasing evidence suggests that fibro-blast growth factors (FGFs), a family of signaling morphogens, function as axon guidance molecules as well, directing the navigation of growing axons in diverse environments [3]. FGFs in the nervous system are essential for early morphogenesis, neurogenesis, and maintaining neuronal physiological homeostasis [4]. However, FGFs and their receptors (FGFRs) are still widely expressed at later stages of the development, even after the full patterning of the central nervous system. Studies demonstrate that these persistent FGFs continue to play critical roles in axon guidance, either directly or indirectly ensuring axons extend along their correct trajectories [5]. For example, FGF8 secreted from the midbrain–hindbrain boundary (MHB) initially regulates the ordered specification of neuronal subtypes, thereby mediating MHB regional patterning [6]. Then, at later developmental stages, MHB-FGF8 regulates the pathway selection of various axons, directly attracting trochlear motor axons, or indirectly repelling retinal ganglion cell (RGC) axons and midbrain dopaminergic neuron (mDAN) axons [3]. However, compared with the research on the chemo-attractive effects of FGFs, the axonal repellent effects of FGFs and their downstream molecular mechanisms urgently need to be further explored.

The thalamus serves as a critical relay station for sensory processing, integrating and transmitting diverse peripheral sensory signals (excluding olfactory inputs) to the cerebral cortex via thalamocortical axons (TCAs). These signals are relayed through thalamic neurons before projecting to cortical targets [7,8]. Current nomenclature distinguishes the prethalamus (ventral thalamus, anterior to the zona limitans intrathalamica [ZLI]) from the thalamus (dorsal thalamus, posterior to the ZLI). During development, TCAs originating from the thalamus extend to the mantle surface of the prethalamus. Upon approaching the hypothalamus, they abruptly turn dorsally to enter the internal capsule, ultimately navigating to distinct cortical regions [7]. Studies have revealed a repulsive effect of the high concentration of FGF3 in the hypothalamus, which not only rejects hypothalamic H-NH (hypothalamo–neurohypophyseal) axons, but also guides TCAs to avoid the hypothalamus and make a sharp lateral turn into the ventral telencephalon [9,10]. However, the downstream mechanisms of how morphogenetic FGFs act on TCAs directly or indirectly remain poorly understood. Therefore, this study aims to use chick thalamic TCAs as a model to investigate the downstream repulsive molecular mechanisms of FGF3.

To investigate this, we used pharmacological inhibitors to block FGFR signaling pathways in vitro and in vivo to examine the direct and indirect TCA repulsive effects of FGF3. FGFs exert their effects through receptor tyrosine kinases known as FGF receptors, which initiate intracellular signaling cascades upon ligand binding. FGF downstream signaling pathways involved in the axon guidance mainly include the mitogen-activated protein kinase (MAPK) pathway, phosphoinositide 3-kinase (PI3K) pathway, phospho-lipase C-gamma (PLCγ) pathway, and non-canonical Phosphatidylcholine-specific phospholipase C (PC-PLC) pathway [3,11]. Among these four downstream pathways, our results indicate that FGF3 selectively utilizes the PC-PLC pathway to directly stimulate asymmetrical repellent growth of developing TCAs. Furthermore, we investigate the FGF-dependent control of the repellent molecule *Slit1* through expression analyses and pharmacological studies. Here we identify that FGF3 regulates *Slit1* expression by employing the PI3K-Akt downstream signaling pathway. Together, these results establish that two distinct FGFR downstream pathways—PC-PLC and PI3K-Akt—orchestrate TCA guidance through direct and indirect mechanisms, respectively, ensuring precise trajectory formation during thalamocortical circuit assembly.

## 2. Results

In the development of the central nervous system, FGFs exhibit dose-dependent dual functions, among which the repulsive effect of FGFs plays an important role in establishing complex neural network systems. Previous experiments confirmed that high concentrations of FGF3 repel TCAs into the ventral telencephalon, ensuring precise sensory pathway formation [10]. Therefore, based on previous phenotype descriptions, we combined the direct and indirect axonal rejection effects of FGF3, using chick TCAs as a model to further detect the downstream guidance pathways of FGF3 on TCA rejections.

### 2.1. PC-PLC Pathway Mediates the Direct TCA Chemorepulsion of FGF3

**Figure 1:**
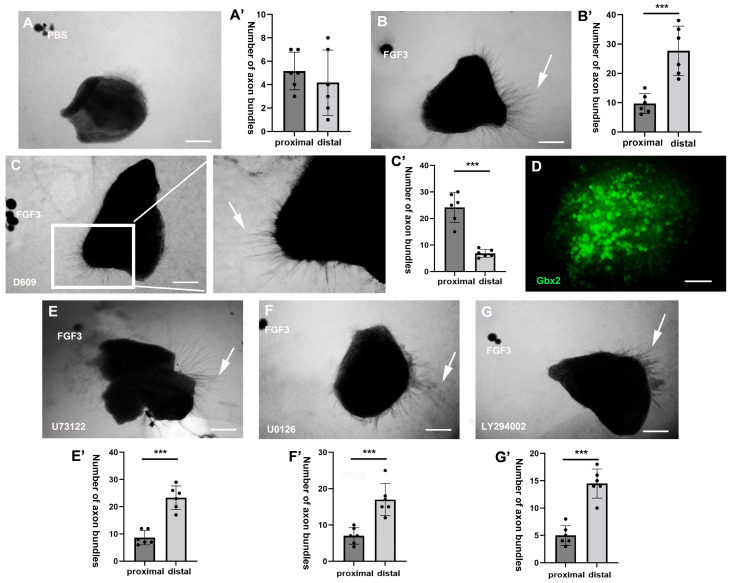
Pharmacological inhibition of PC-PLC pathway affects the TCA-repulsive effect of FGF3. Thalamic explants were co-cultured with FGF3 beads (500 ng/mL), and inhibitors of different pathways were added to the culture medium (n ≥ 6 in each group). (**A**–**C’**) Compared with the control groups (**A**,**A’**: negative PBS control; **B**,**B’**: positive FGF3 control), when cultured with PC-PLC inhibitor D609 (10 µM), FGF3 beads lose their ability to repel TCA axons, even exhibiting attractive effects, since more thalamic axons (arrow) were observed on the proximal side (towards FGF3 beads) (**C**,**C’**). (**D**) The expression of glutamatergic marker *Gbx2* in thalamic explants. (**E**–**G’**) The inhibitors of U73122, U0126, and LY294002, however, show little effects on the TCA axonal rejection of FGF3. Scale bars (**A**–**C**,**E**–**G**) = 80 µm; ***: *p* < 0.001; Scale bar (**D**) = 40 µm.

To determine the FGFR downstream signaling pathways mediating the direct repellent effect of FGF3, PI3K, MAPK, PC-PLC, and PLCγ pathway inhibitors were applied to the three-dimensional co-culture system containing FGF3 beads (500 ng/mL) and thalamic explants (E3–4). The usage of inhibitors (10 µM) was as follows: PI3K inhibitor, LY294002; MAPK inhibitor, U0126; PC-PLC inhibitor, D609; and PLCγ Inhibitor, U73122. After 48 h of culture, TCA growth was detected microscopically. The preliminary experimental results showed that compared with PBS negative controls (Figure 1A,A’) and FGF3 positive controls (Figure 1B,B’), the PC-PLC pathway inhibitor D609 significantly hindered the repellent effect of FGF3 (Figure 1C,C’). There were significantly more TCA axons in the proximal (towards FGF3 beads) section than axons in the distal (away from FGF3 beads) section (Figure 1C’). Thalamic explant identity was confirmed by robust expression of the thalamic marker *Gbx2* (Figure 1D). In contrast, the other three pathway inhibitors had no effect on TCA guidance (Figure 1E–G’): the number of thalamic axons was significantly greater in the distal section (away from FGF3 beads) than in the opposite section. These preliminary results suggest that the PC-PLC pathway may be essential for FGF3-dependent repulsion, enabling TCAs to shift away from the FGF3+ hypothalamus, then enter towards and navigate into the internal capsule.

**Figure 2:**
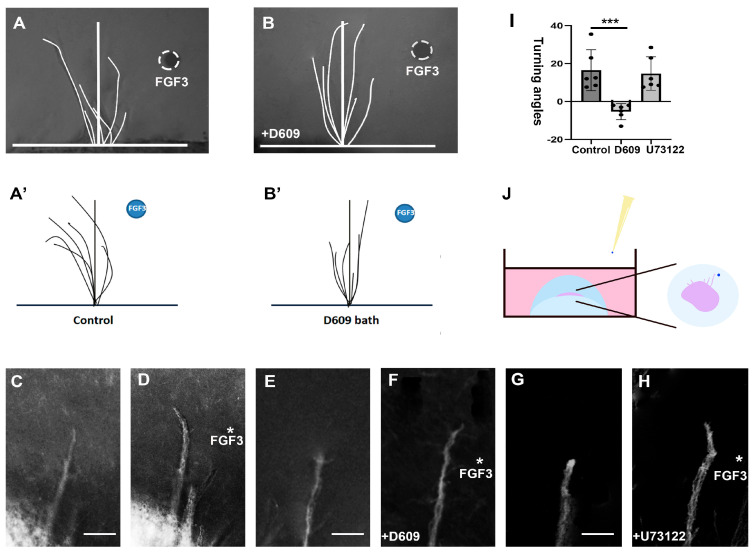
FGF3 induces the repulsion of TCA growth cones by PC-PLC signaling pathway. (**A**–**B’**) A FGF3 bead placed 25–50 µm away from the growth cone extending from a 24 h old thalamic explants for 3 h, with or without inhibitor (such as D609) in the media. (**C**,**D**) Phase micrographs of a representative growth cones in control media at *t* = 0 min (**C**), and after 3 h in the presence of an FGF3 gradient (**D**). (**E**,**F**) Trajectories of growth cones at *t* = 0 min (**E**), and at *t* = 3 h in 10 µM D609-supplemented media (**F**). (**G**,**H**) Growth cones prior to applying the FGF3 beads (*t* = 0 min) in control media (**G**), and growth cones 3 h after continuous exposure to FGF3 in the presence of 10 µM U73122 (**H**). (**I**) Differences in mean turning angles for distinct groups. (**J**) A schematic diagram showing the placement of FGF3 beads to the growth cones. ***: *p* < 0.001; asterisk: the implanation of FGF3 beads; scale bars = 25 µm.

To validate the role of the PC-PLC pathway in FGF3-mediated TCA guidance, we conducted growth cone turning assays. After a 24 h cultivation of the thalamic explants (E4), Affi-Gel Blue beads adsorbed with high concentration FGF3 (500 ng/mL) were placed on one side of an individual axon bundle to establish a high localized concentration FGF3 gradient (Figure 2A). A distinct pathway inhibitor, such as D609 (10 μM), was applied to the media of the co-culture system (Figure 2B) for 3 h. In control conditions, the bead containing FGF3 exerted the repellent effect on TCA growth cones in the control medium (Figure 2C,D). However, the growth cones did not repel from the FGF3 source when PC-PLC inhibitor D609 (10 μM) was present in the bathing solution (Figure 2E,F). The mean turning angles for the FGF3 positive controls and PC-PLC inhibition were as follows: FGF3 positive control, θ = 16.52 ± 10.80 (SD), n = 6; D609, θ = −5.33 ± 4.23 (SD), n = 6. Results for the inhibitor D609 baths were significantly different (*p* < 0.001, unpaired two-tailed Student’s *t* test) compared with the turning angles measured in the FGF3 control bath. It has been reported that PLCγ inhibitor U73122 is required to block FGF2-stimulated axon repulsion of RGC growth cones [12]. In contrast, in our results, U73122 had no effect on the FGF3-induced chemorepulsion at a concentration of 10 μM (Figure 2G,H). The mean turning angles for PLCγ inhibition were as follows: θ = 14.77 ± 8.81 (SD), n = 6. The statistical analyses between positive controls and U73122 groups revealed no significant differences (*p* > 0.05).

### 2.2. PC–PLC Pathway Is Necessary for TCA Repulsion In Vivo

**Figure 3:**
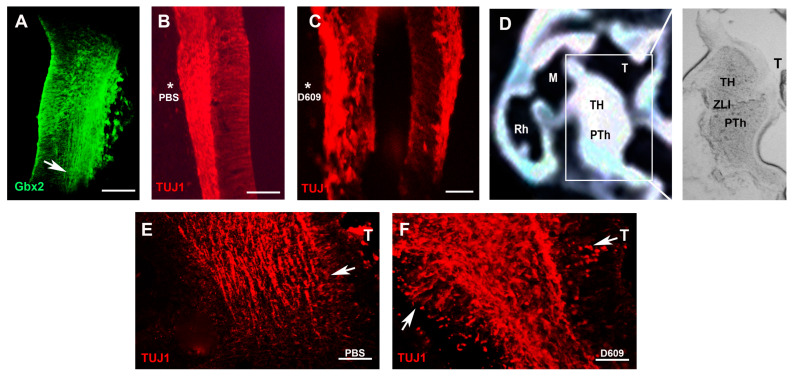
The PC-PLC pathway is necessary for TCA extension in vivo. (**A**) The expression of thalamic glutamatergic marker Gbx2 in a transverse thalamic section at E5. (**B**) In a control bead implant (asterisk), transverse sections show thalamic pioneer projections at E5. (**C**) After a D609 bead implantation (asterisk) for 2 days, pioneer projections in the thalamus appear to be disorganized at E5. (**D**) Schematic showing a sagittal view of the E5 forebrain; the right-hand panel shows a high-power view of the boxed region. (**E**) PBS controls show that thalamic TUJ1+ axons (arrow) project towards the E5 prethalamus in a sagittal view. (**F**) With the addition of D609, the extension of thalamic axons is chaotic, and some axons (arrows) are outside the axonal bundle. TH, thalamus; PTh, prethalamus; M, midbrain; Rh, rhombencephalon; T, telencephalon; ZLI, zona limitans intrathalamica. Scale bars (**A**–**C**) = 50 µm; scale bars (**E**,**F**) = 25 µm.

To validate the role of the PC-PLC pathway in high-concentration FGF3-mediated TCA repulsion in vivo, we analyzed the pathfinding of TCAs following pharmacological inhibition. Thalamic TCAs were visualized using antibodies against *TUJ1* (a marker of class III β-tubulin in neuronal precursors and axons) and *Gbx2* (a transcription factor specific to thalamic glutamatergic neurons and their projections). At E5, the labeling of *Gbx2* expression revealed the growing TCAs extending ventrally towards the hypothalamus at the mantle zone (Figure 3A). To perturb PC-PLC signaling, Affi-Gel beads loaded with the PC-PLC inhibitor D609 (10 µM) or PBS (control) were implanted adjacent to the thalamus in E3 embryos and incubated for 48 h. Axon trajectories were assessed using transverse (Figure 3A–C) and sagittal (Figure 3D–F) sections. In transverse sections of PBS-treated embryos (asterisk), the expression of *TUJ1*+ thalamic axons was detected within the mantle zone (Figure 3B). In contrast, embryos implanted with D609 beads (asterisk) exhibited disrupted axonal organization: thalamic axons appeared scattered and randomly oriented compared with the contralateral (untreated) side (Figure 3C).

To further assess the role of PC-PLC in TCA guidance in vivo, we analyzed sagittal sections of embryos implanted with PBS or D609 beads adjacent to the thalamus (Figure 3D). In the PBS controls, TCAs extended ventrally from the thalamus toward the prethalamus (Figure 3E). D609 inhibition, however, caused chaotic axonal bundle growth with aberrant axon turning (Figure 3F). This disruption of TCA fasciculation and trajectory confirms that the PC-PLC pathway is essential for FGF3-mediated chemorepellent signaling in vivo. Thus, inhibition of the in vivo PC-PLC pathway alters the growth trajectory of endogenous TCAs.

### 2.3. FGF3 Signaling Regulates the Expression of Slit1

**Figure 4:**
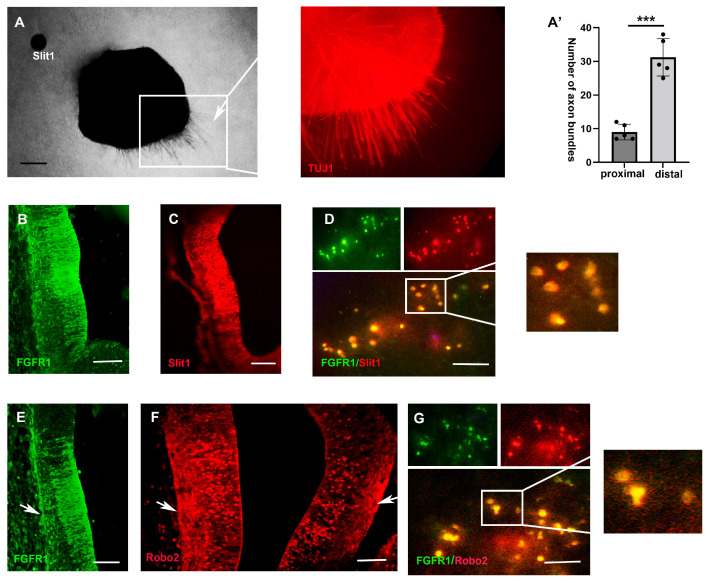
Expression patterns of *Slit1* and downstream *Robo2/FGFR1* genes in the E5 chick diencephalon. (**A**,**A’**) *Slit1* beads show repellent effects on thalamic axons, and the right-hand panel shows *TUJ1* expressions on thalamic axons of the boxed area. There are significantly more axon bundles from distal faces of thalamic explants (away from *Slit1* beads) (n = 6 explants, *** *p* < 0.0001). (**B**,**C**) Immunofluorescence shows *FGFR1* and *Slit1* expressions in the diencephalon. (**D**) Double-labelled analyses showing co-expressions (orange) of *Robo2* and *FGFR1*. (**E**,**F**) Comparison of *Robo2* (**E**) and *FGFR1* (**F**) expressions in the developing diencephalon and in TCAs (arrows). (**G**) Double-labelled analyses showing co-expressions (orange) of *Robo2* and *FGFR1*. ***: *p* <0.001; scale bars (**A**–**C**,**E**,**F**) = 50 µm; scale bars (**D**,**G**) = 40 µm.

Slit1, a glycoprotein acting via Roundabout (Robo) receptors, mediates developing axons and migrating cell repulsion [13]. During TCA pathfinding, *Slit1* expressed in the rat hypothalamus and ventral thalamus directs TCAs from the diencephalon, extending into the ventral telencephalon [14]. To validate the chemorepellent role of the classic axon-repellent Slit1 in chick TCAs, Slit1 beads and E4 chick thalamic explants were cultured in three-dimensional collagen gel. After 48 h, Slit1-soaked beads repelled TCA trajectories, resulting in significantly higher *TUJ1*+ axon density in distal explant regions (Figure 4A,A’). Consistent with previous findings, when TCAs were projecting towards the hypothalamus, *FGFR1* was expressed in both the hypothalamus and thalamus regions at E5 (Figure 4B,E). Immunofluorescence revealed robust *Slit1* expression in the dorsal hypothalamus during TCA pathfinding (Figure 4C). To further investigate whether there is an intersection between the FGFR pathway and the expression of Slit, we examined the co-expression of *FGFR1* and *Slit1* in the ventral diencephalon. Here, the immunoassay results showed the co-expression of *FGFR1* in the *Slit1*+ ventral diencephalic cells, suggesting FGFR signaling in regulating hypothalamic *Slit1* expressions (Figure 4D).

We found that Slit1 receptor Robo2 was widely expressed in the thalamus (Figure 4F). Its expression overlapped spatially with *FGFR1* (Figure 4E), and both proteins localized to the mantle zone where TCA projections reside (Figure 4E,F). Furthermore, there was an overlapped expression of *FGFR1* and *Robo2* at the cellular level in the thalamus (Figure 4G), indicating potential synergy between the FGFR1 and Slit1 pathways in the pathfinding of TCAs.

**Figure 5:**
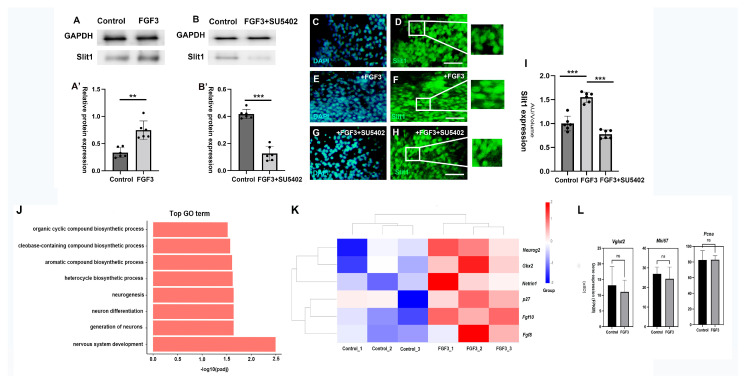
FGF3 enhances the expression of repellent molecule *Slit1*. (**A**,**A’**) Western blot analyses showed that, compared with blank controls, FGF3 (300 ng/mL) in the culture media significantly increased the expression of *Slit1* (n = 6). (**B**,**B’**) In contrast, the FGFR inhibitor SU5402 (20 µM) reduced the expression of the repellent factor *Slit1* (n = 6). (**C**–**H**) Enhanced immunofluorescence staining of DAPI (blue) and Slit1 (green) shows FGF3 upregulates *Slit1* expression: explants of E4 diencephalon were cultured either for two days alone (**C**,**D**), with FGF3 (**E**,**F**), or with FGF3+SU5402 in the media (**G**,**H**). (**I**) Quantification of fluorescent signal from immuno-labelled Slit1 per volume of the thalamic explant. (**J**) Top GO items of RNA-seq were shown according to the gene counts. (**K**) Heatmap visualizes mRNA expression patterns of relative genes across different groups (controls and FGF3-treated). (**L**) Bar graphs for specific genes at mRNA transcription level (*Vglut2*, *Mki67*, *Pcna*), comparing their expression between controls and FGF3-treated explants. ns: Not-significant; FPKM, fragments per kilobase of transcript per million mapped reads; GO, gene ontology; **: *p* < 0.01; ***: *p* < 0.001; Scale bars = 40 µm.

To investigate whether FGF3 indirectly influences TCA guidance, we bisected E4 di-encephalic explants and cultured the halves in collagen gel under two conditions: one was treated with FGF3, and the other contained FGF3 + the FGFR inhibitor SU5402 in the media. After 48 h, Western blot and immunofluorescence analyses revealed that FGF3 robustly upregulated *Slit1* expression (Figure 5A,A’ and Figure S1A), whilst FGF3 failed to increase the expression of *Slit1* when SU5402 was co-administered (Figure 5B,B’ and Figure S1B), suggesting FGFR signaling is necessary for *Slit1* gene expression. Likewise, immunofluorescence imaging demonstrated elevated *Slit1* expression levels in FGF3-treated explants (Figure 5E,F) compared with blank controls (Figure 5C,D). However, the expression levels of *Slit1* were significantly reduced in the presence of FGFR inhibitor SU5402 (Figure 5G–I). These results indicate that FGF3 can enhance the expression of the classical repellent cue *Slit1* via FGFR activation, thereby indirectly guiding the pathway selection of TCAs.

RNA-seq analysis was conducted to explore the genes and signaling pathways regulated by FGF3 in the chick diencephalon. A total of 21,888 genes were analyzed in this study by comparing the gene expression levels between the control groups and the FGF3-treated groups. Gene Ontology (GO) results on the identified DEGs (differentially ex-pressed genes) show that there are major changes in biological processes, particularly those related to nervous system development, neurogenesis, and neuron differentiation (Figure 5J). RNA-seq analysis revealed that FGF3 treatment upregulated the transcriptional level of *Neurog2*, *Gbx2* and *p27* in diencephalic explants, indicating that FGF3 is associated with glutamatergic neurogenesis and differentiation (Figure 5K). However, the expression levels of mature glutamatergic marker gene *Vglut2* and proliferative marker (*Mki67*, *Pcna*) in control and FGF3-treated explants shows no significant differences (Figure 5L). Interestingly, FGF3 affects the mRNA level of *Fgf10* and *Fgf8*, and simultaneously increase the expression of canonical *netrin-1* (Figure 5K), suggesting a synergic effect between FGF3 and other key molecules in the diencephalon.

### 2.4. Indirect Repulsive Effects of FGF3 on TCAs Is Induced by the PI3K Downstream Pathway

**Figure 6:**
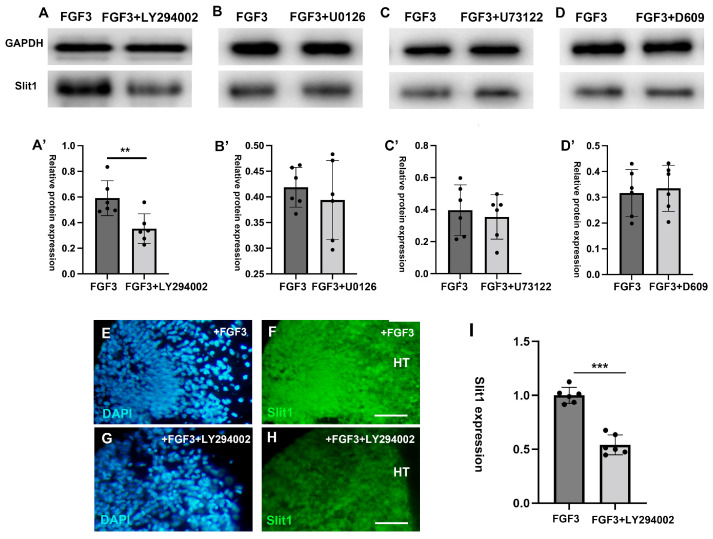
PI3K downstream signaling is required for *Slit1* expression in the diencephalon. Western blot analyses of *Slit1* expression in the diencephalic explants on the second day post the addition of different pathway inhibitors (**A**,**A’**). Compared with FGF3 positive controls, PI3K pathway inhibitor LY294002 significantly reduced the expression of *slit1* (**B**–**D’**). However, the other pathway inhibitors U0126, D609 and U73122 had no significant effect on the expression of *slit1* (**E**–**H**). Immunofluorescence staining of DAPI (blue) and *Slit1* (green) in the diencephalic explants on the second day post the addition of FGF3 (**E**,**F**), or post the addition of FGF3+LY294002 (**G**,**H**). (**I**) Quantification analyses of *Slit1* per volume of the thalamic explant. **: *p* < 0.01; ***: *p* < 0.001; scale bars = 80 µm; HT: hypothalamus.

To identify the downstream FGFR pathway responsible for regulating *Slit1* expression in the diencephalon, we cultured E4 diencephalic explants in collagen gel with specific downstream inhibitors targeting distinct FGFR-signaling cascades (PI3K, MAPK, PLCγ, and PC-PLC) and FGF3. After 48 h, Western blot analysis quantified the ex-pression of *Slit1* to assess which inhibitor blocked FGF3-mediated induction. In contrast to FGF3 positive controls, the PI3K inhibitor LY294002 significantly attenuated *Slit1* expression (Figure 6A,A’ and Figure S2A,A’). In contrast, inhibitors targeting the MAPK/ERK (U0126), PLCγ (U73122), or PC-PLC (D609) pathways showed no obvious effect on *Slit1* expression levels (Figure 6B–D’ and Figure S2B–D’). Consistent with these Western results, immunofluorescence results showed that, while FGF3 robustly elevated *Slit1* in controls (Figure 6E,F), PI3K inhibitor LY294002 antagonized the effect of FGF3 and reduced the expression levels of *Slit1* (Figure 6G–I and Figure S3A,B). Collectively, FGF3 upregulates *Slit1* expression in the diencephalon, specifically via the PI3K pathway downstream of FGFR1, implicating this mechanism in the indirect regulation of TCA guidance.

## 3. Discussion

The thalamus, a critical sensory hub in the central nervous system, is implicated in cerebral hemorrhage, neurodegeneration, and cerebrovascular disorders. Dysregulated FGFR1 signaling is implicated in cortical malformations and schizophrenia risk [15]. Slit/Robo mutations are linked to ASD (autism spectrum disorder) and intellectual disability [16], whilst PLC is associated with schizophrenia and depression, highlighting conserved roles in neural connectivity [17]. Thalamocortical circuit disruptions further underpin neuropsychiatric conditions such as epilepsy, emphasizing the need to decode thalamic developmental mechanisms for therapeutic advances in neural repair [7].

FGF3, a member of the evolutionarily conserved FGF7 subfamily of morphogenes, directs sensory network formation during late embryogenesis via repulsive guidance. At high concentrations, FGF3 repels thalamic sensory TCAs, guiding them away from the hypothalamus and facilitating a sharp lateral turn into the ventral telencephalon [10]. While FGF-mediated axon repulsion is well studied in RGC axons, H-NH axons and midbrain dopaminergic (mDAN) axons, the intracellular downstream molecular mechanisms of FGFs underlying thalamic TCA chemorepulsion remain unclear [9,18,19]. Therefore, the primary objective of this study is to further investigate the specific intracellular downstream signaling pathways of FGF3, as a repellent directly or indirectly regulating TCA guidance.

### 3.1. Direct Axonal Repellent Effects of FGFs Through the PC-PLC Pathway

Axonal growth cone responses to FGF signals are highly dynamic, varying across spatial and temporal contexts. Current evidence identifies MAPK, PLCγ, PC-PLC, and PI3K as key downstream pathways mediating FGF-dependent axon guidance and navigation [3,12,20]. RGC axon pathfinding is a well-established model for studying axonal development. By using in vivo and in vitro analyses, the growth cone rejection induced by FGF2 is mediated by FGFR downstream PC-PLC and PLCγ signaling pathways [12,21]. Notably, the PC-PLC pathway has also been implicated in TCA branching in murine models [22]. In our study, in vitro experiments revealed that, among four pathway inhibitors, only PC-PLC inhibitor D609 disrupted FGF3-induced growth cone turning. Meanwhile, in vivo studies confirmed the necessity of the PC-PLC pathway for TCA pathfinding. Together, in vitro and in vivo data suggest that this re-steering effect of high-concentration FGF3 on TCAs operates exclusively through the PC-PLC pathway.

Phosphatidylcholine-specific phospholipase C (PC-PLC) hydrolyzes membrane phosphatidylcholine (PC) to mediate the production of phosphocholine (PCho) and non-phosphatidylinositol 4,5-bisphosphate (PIP2)-derived diacylglycerol (DAG). However, mammalian PC-PLC has not been cloned; sequenced and structural information is unavailable [23]. In contrast, phospholipase C-gamma (PLCγ) catalyzes the hydrolysis of PIP2 into DAG and inositol-1,4,5-triphosphate (IP3) [24]. Hence, the mechanism underlying axonal repulsion in both pathways may involve the suppression of the functional activities of the downstream co-product, DAG. FGF3 in the chick hypothalamus acts as a chemorepellent to steer prethalamic GABAergic axons away from their source, ensuring their correct trajectory during development [25]. Similarly, basic fibroblast growth factor (bFGF) regulates rat cortical precursor proliferation via thalamic-derived mitogenic activity [26], while chick hypothalamic FGF3/FGF10 functions as a chemoattractant on TCAs during late embryogenesis. Notably, FGF3/FGF10 exhibits a dual concentration-dependent mechanism: low concentrations induce chemoattraction during early TCA development, whereas high concentrations induce repulsion at later stages [10,27]. Despite these insights, the specific downstream pathway mediating FGF3’s guidance effects has not been resolved. In addition, PLCγ drives FGF effects in regulating chick ciliary ganglion neurite outgrowth [28], and DAG-dependent cytoskeletal remodeling in *C. elegans* [29,30,31]. Nevertheless, zebrafish FGF3 mutants and transgenic fish expressing a dominant negative FGFR1 in hypothalamic neurons lack neurohypophysis innervation [32]. However, in the diencephalon, there are few reports on the intracellular downstream mechanisms of direct axonal rejection of FGFs in other regions. Here, our results indicate that the PC-PLC pathway (but not the PLCγ pathway) is the mediator of FGF3-induced TCA repulsion, indicating that the PC-PLC pathway may not operate through a shared DAG pathway with PLCγ, but acts through another independent pathway. Instead, PC-PLC most likely exerts its effects via its other enzymatic product, PCho, which has been shown to mediate repulsive neurite guidance [33]. Alternatively, differences in these PLC pathways may occur at downstream terminals, such as the GTPases Rho family that regulates cytoskeletal actin dynamics [34]. In addition, the Rho family member Rac1, linked to FGFR signaling [35], controls axon retraction in *Drosophila* [36]. While FRS2-Ras-MAPK is universal, PLCγ/PC-PLC activation exhibits cell-type specificity [3,4], potentially explaining PLCγ’s absence in developing TCAs.

These results emphasize the need to define how PC-PLC interacts with cytoskeletal effectors (e.g., Rho GTPases) to resolve its unique guidance role.

### 3.2. FGF3 Induces Slit1 in the Diencephalon

Emerging evidence indicates that FGFs can indirectly guide axonal pathway selection by regulating certain classical guidance factors. For example, in *Xenopus* retinal axons, FGF signaling sustains *Slit1* and Semaphorin3a (Sema3a) expression to repel growth cones towards the optic tectum, with FGFR1 exclusively driving *Slit1* and FGFR2-4 controlling Sema3a [18,37,38]. Similarly, *Slit1* in the rat dorsal hypothalamus serves as a chemorepellent, directly repelling TCAs into the ventral hypothalamus [14], while the other family member FGF8 is associated with *Slit1* expression during Xenopus appendage regeneration [33]. These guidance systems are evolutionarily conserved: *Slit1*’s role in thalamocortical patterning is shared across avian and mammalian models [39]. However, there is currently no evidence to prove whether FGFs expressed in the hypothalamus could affect the expression of *Slit1* in the hypothalamus, indirectly affecting the pathway selection of TCAs via FGFR signaling. FGF3 binds both FGFR1 and FGFR2, though FGFR1 exhibits lower ligand-dependent activation compared with FGFR2 [40]. Studies have demonstrated that FGFR1 can be widely expressed in neurons, astrocytes, and radial glia in the brain [3,41,42] and that it drives *Slit1* expression in Xenopus renal epithelia [37]. Additionally, some studies have reported that FGFR1 and Slit1 receptor Robo2 are significantly expressed in the thalamus [43,44], yet prior studies lacked evidence of their co-localization. Consistent with these findings, we demonstrate that *FGFR1* and *Slit1* are co-expressed in the ventral diencephalon, suggesting FGFR signaling may regulate hypothalamic *Slit1* levels. Moreover, FGFR1 and Slit1 receptor Robo2 are widely co-expressed in the thalamus, indicating potential collaboration between both pathways in directing TCA polarity. We also found that exogenous FGF3 upregulated *Slit1* expression in vitro. Furthermore, SU5402, a FGF receptor tyrosine kinase inhibitor [45], abolished the induction of *Slit1* expression induced by FGF3. Together, these findings indicate that FGF3 indirectly guides TCA growth polarity by inducing the expression of *Slit1* through FGFR1 signaling.

### 3.3. Contribution of PI3K Signaling to Indirect TCA Axon Guidance

Axonal turning at critical choice points is not governed by a single type of guidance cue but emerges from the collaboration of multiple signaling pathways. Therefore, to demonstrate that FGFs could collaborate with the Slit pathway to regulate TCAs’ pathfinding, we conducted in vitro assays targeting candidate FGFR downstream pathways (predominantly the MAPK pathway). However, inhibition of the MAPK pathway shows no effect on diencephalic *Slit1* expression levels, and the expression of *Slit1* is more likely involved in the other FGFR signaling pathway(s).

The FGF-mediated PI3K signaling pathway regulates the co-expression of *Slit1* and Se-ma3a through activation of FGFR1 and FGFR2, respectively, ensuring precise RGC axon pathfinding [18]. Then, how does FGF3 induce *Slit1* expression in the chick diencephalon? Consistent with these studies, we find that PI3K pharmacological inhibitor LY294002, instead of inhibitors of MAPK, PLCγ, or PC-PLC pathways, significantly reduces *Slit1* expression level in the diencephalon. The PI3K pathway is well known to cooperate downstream of extrinsic guidance cues [46]. This suggests that PI3K probably acts downstream of FGFRs to maintain hypothalamic *Slit1* expression. In support of this, the TCA axonal phenotype observed with PI3K inhibition resembles that observed with FGFR inhibition SU5402.

The PI3K signaling pathway regulates neuronal development via Akt phosphorylation [47]. Here, PI3K-AKT activation could upregulate *Slit1* expression, indirectly repelling TCAs. Pharmacological PI3K inhibition (LY294002) probably suppresses phosphorylation of PI3K (p-PI3K) and AKT (p-AKT), inhibiting *Slit1* expression and Slit1-dependent axon repulsion.

**Figure 7:**
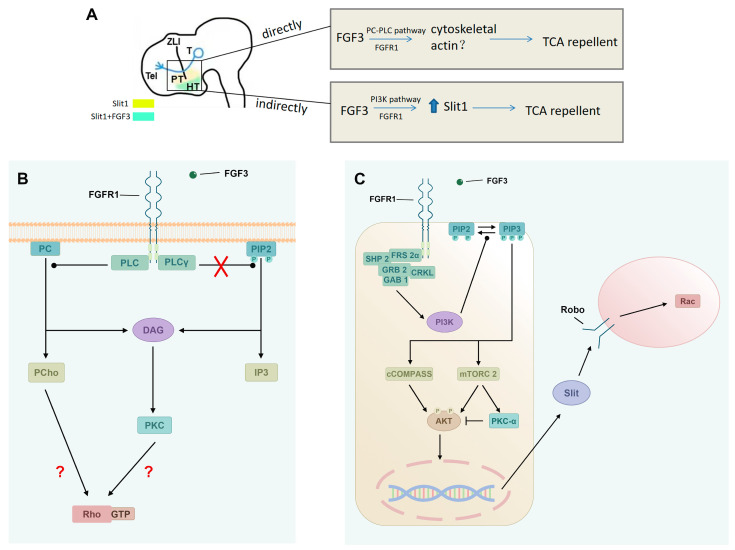
Working model of FGF3 on the guidance of TCAs in the thalamus. (**A**) FGF3 acts directly to regulate the turning of dorsal TCAs, which may be achieved by regulating the reorganization of PC-PLC downstream cytoskeletal actin. FGF3 is enriched in the ventral hypothalamus, potentially affecting expression levels of *Slit1* activity by the PI3K pathway, and thus indirectly regulates the pathfinding of TCAs. (**B**) Schematic details the PC-PLC pathway activated by FGFR1 and FGF3, depicting components like PLC, DAG, IP3, PKC, and unresolved links to Rho and GTP. (**C**) Schematic depicts the indirect PI3K pathway, where FGFR1/FGF3 trigger signaling through molecules like SHP2, GRB2, etc., ultimately affecting *Slit1*, and cytoskeleton regulatory protein Rac. TH: thalamus; PT: prethalamus; HT: hypothalamus; T: telencephalon; ZLI: zona limitans intrathalamica; FGF: fibroblast growth factor; FGFR: fibroblast growth factor receptor; PC: phosphatidylcholine; PLC: phospholipase C; PLCγ: phospholipase C gamma; PI3K: phosphoinositide 3 kinase; PIP2: phosphatidylinositol 4,5-bisphosphate; DAG: diacylglycerol; IP3: inositol 1,4,5-trisphosphate; PCho: phosphocholine; PKC: protein kinase C; SHP2: Src homology 2 domain-containing protein tyrosine phosphatase 2; FRS2α: fibroblast growth factor receptor substrate 2 alpha; GRB2: growth factor receptor-bound protein 2; CRKL: CRK-like proto-oncogene; GAB1: Grb2-associated binding protein 1; cCOMPASS: catalytically active COMPASS; mTORC2: mammalian target of rapamycin complex 2; AKT: protein kinase B; Robo: Roundabout; red ×: the assumed pathway does not exist; red ?: probable pathways.

In summary, this study provides the first comprehensive analysis of the downstream pathways mediating FGF3’s direct and indirect repellent effects on TCAs (Figure 7). Pharmacological blockade confirms FGFR downstream signaling in TCA axons. Both in vivo and in vitro data identify that the PC–PLC pathway is the transduction mechanism directing FGFR signaling in repulsive TCA turning. In addition, we reveal a novel extrinsic FGFR1-PI3K-Slit1 axis in the developing diencephalon. Future work should explore whether other axon guidance cues are similarly controlled by FGFs, and the identity of FGFR1’s downstream transcription factors that control the *Slit1* expression. Moreover, genetic and molecular approaches specific to FGFR signaling in TCA guidance will be needed in the future.

## 4. Materials and Methods

### 4.1. Explant Culture

Fertilized eggs of local Brown chicks were obtained Jiangxi Breeding Factory. Chick embryos were staged and treated with Dispase (1 mg/mL, Roche, Mannheim (Baden-Württemberg), Germany) for isolation, whilst the diencephalon (E4, embryonic day 4) were dissected out in an ‘open book’ configuration. Explants of the thalamus were dissected. Explants were cultured in 3-dimensional collagen gels (R&D #344010001, Minneapolis, MN, USA) based on published techniques [48]. Affi-Gel Blue gel beads (BioRad #153-7302, Hercules, CA, USA) were washed 3 times in PBS and then soaked in FGF3 protein (R&D) overnight at 4 °C prior to culture. FGF3 protein was used, based on standards, at concentrations of 300 ng/mL or 500 ng/mL; its antagonist SU5402 (MedChemExpress, Monmouth Junction, NJ, USA) was used at 20 µM. *Slit1* (R&D) protein was used at concentrations of 200 ng/mL [49]. According to the requirements for distinct experiments, different inhibitors were added to the control solution. Based on our initial research results and published data [12,50,51], several different pharmacological inhibitors (10 µM, MedChemExpress) were added in vitro or in vivo, including the PI3K inhibitor, LY294002; the MAPK inhibitor, U0126; the PC-PLC inhibitor, D609; and the PLCγ Inhibitor, U73122. Control solutions contained the same concentration of dimethyl sulfoxide (DMSO).

### 4.2. Immunofluorescence Analyses

Immunohistochemical analyses of embryos (n ≥ 6) and explants (n ≥ 6) were performed according to standard whole-mount or cryostat sectioning techniques [52]. In this study, the following antibodies were used: anti-*Tuj1* (anti neuron-specific class IIIβ-tubulin; ab 78078, Abcam, Waltham, MA, USA), anti-*FGFR1* rabbit polyclonal (bs-0230R, Bioss, Beijing, China), anti-*Slit1* (bs-11487R, Bioss), anti-*Robo2* (bs-7921R, Bioss), and anti-*Gbx2* (bs-11849R, Bioss, China). Secondary antibodies were conjugated to Alexa 594 or Alexa 488 (Abcam).

### 4.3. Bead Implantation

Affi-Gel Blue beads (Biorad) were pre-soaked in D609 (20 µM) [12] for 24 h at 4 °C. E3 embryos were windowed, and D609-loaded beads were put above the area of the thalamus. The eggs were resealed and incubated for 48 h prior to fixation and analysis.

### 4.4. Western Blot Analysis

After dissecting out the diencephalon, the E4 diencephalic explant was isolated from the middle and bisected into two symmetrical halves. According to different experimental requirements, the bisected explants were treated differently and cultured for 48 h. For protein isolation, embryonic explants were homogenized using a whole protein extraction kit (KGB5303-100, KeyGENBioTECH, Changchun, China). The total protein concentration in the supernatants was estimated using a bicinchoninic acid (BCA) protein detection kit (KGA902, KeyGENBioTECH). Western blot analysis was performed as described previously [53], using the following antibody dilutions: anti-GAPDH (KeyGENBioTECH; 1:2000), and anti-*Slit1* (bs-11487R, Bioss). Band intensities were quantified using Gel-Pro32 software 4.0.

### 4.5. RNA-Seq of Chick Diencephalon

For RNA sequencing, we employed E4 explants of the diencephalon. Samples were divided into a control group and an FGF3-treated group. The control groups consisted of three replicates (Control_1, Control_2, Control_3), and the FGF3-treated group also had three replicates (FGF3_1, FGF3_2, FGF3_3). All the groups were cultured in collagen (with or without FGF3 (300 ng/mL) in the media) for 32 h. Total RNA from the explants was harvested using the RNeasy kit (Qiagen) according to the manufacturer’s instructions. All the RNA-seq data were sequenced by the company (Novogene, Beijing, China). Raw data were firstly processed through fastp software to remove noise and the adaptors. Regarding gene expression, the adjusted *p*-value ≤ 0.05 and |log2 (foldchange)| ≥ 1 (Benjamini and Hochberg’s methods) were set as the threshold of significant differential expressions.

Gene Ontology (GO) enrichment analysis of differentially expressed genes was implemented by the clusterProfiler R package (Version 4.10.0), in which gene length bias was corrected. GO terms with adjusted *p*-value less than 0.05 were considered significantly enriched by differentially expressed genes.

### 4.6. Statistical Analysis

Statistical analyses were carried out using SPSS 27.0 and Prism 9 for PC. Statistical significance of differences in means between groups (n ≥ 6) was determined using a 2-tailed unpaired Student *t* test. *p* values less than or equal to 0.05 indicate a significant difference. Results were reported as the means ± SD.

## Data Availability

Data will be made available upon request.

## Supplementary Materials

The following supporting information can be downloaded at: https://www.mdpi.com/article/10.3390/ijms26157361/s1.
