# Supplementary material for: Direct and Indirect Downstream Pathways That Regulate Repulsive Guidance Effects of FGF3 on Developing Thalamocortical Axons
Source: Int J Mol Sci. 2025 Jul 30;26(15):7361. doi: 10.3390/ijms26157361 (PMC12347462; doi:10.3390/ijms26157361)
Supplement: Supplementary file 1 [file ijms-26-07361-s001.zip › ijms-3499627-supplementary.pdf]

Supplemental Materials

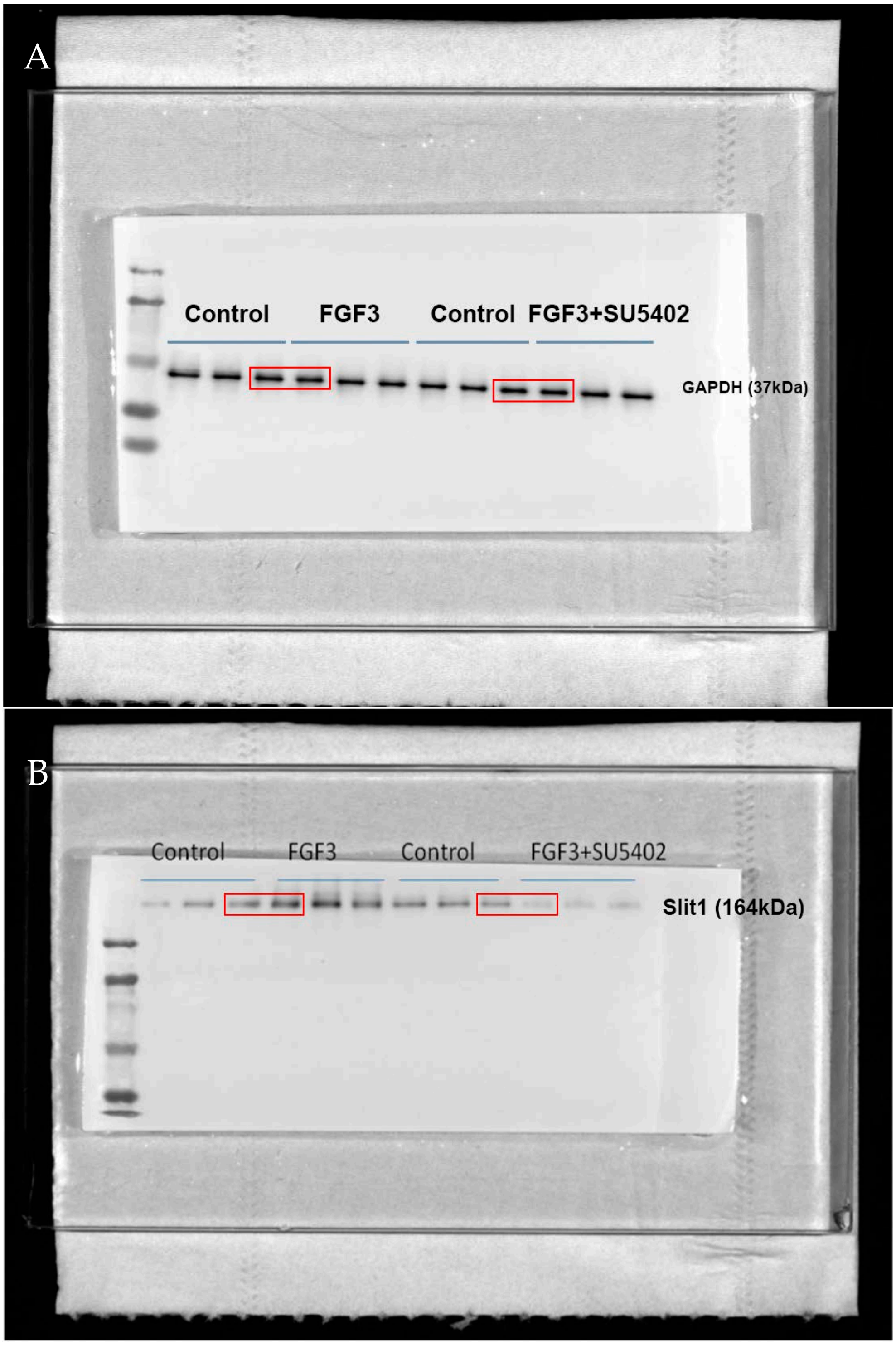

Figure S1. FGF3 upregulates Silt1 protein expression via FGFR signaling. **(A)** Western blot analysis showing GAPDH loading control (37 kDa) under different treatment conditions: Control (untreated), FGF3 stimulation, and FGF3 stimulation combined with the FGFR inhibitor SU5402. (**B**) Western blot analysis showing Silt1 protein levels (detected at ~164 kDa) under different treatment conditions: Control (untreated), FGF3 stimulation, and FGF3 stimulation combined with the FGFR inhibitor SU5402. Red frame: representative wetern blot analysis selected in Figure 5.

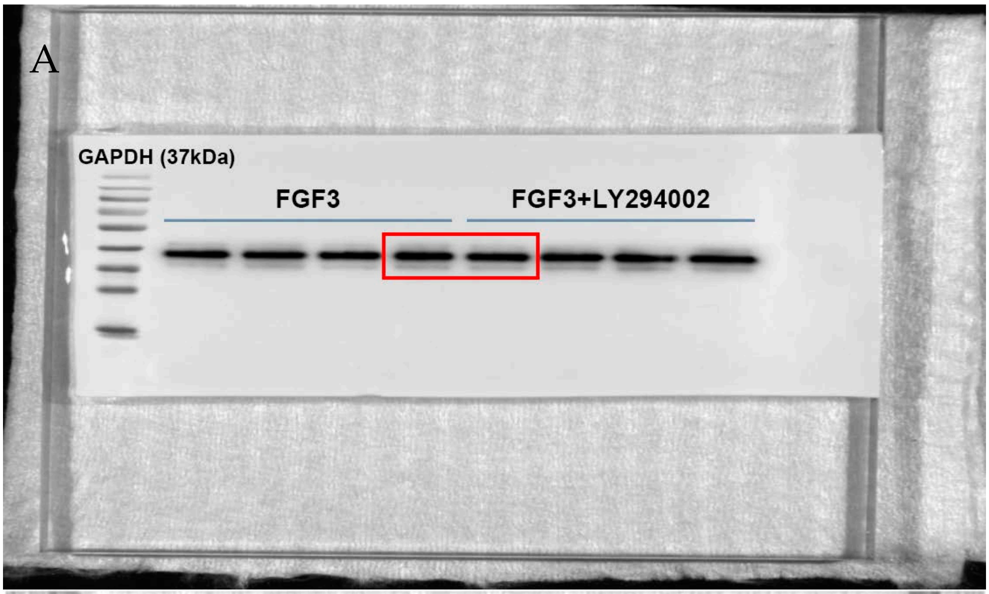
A
GAPDH (37kDa)
FGF3
FGF3+LY294002

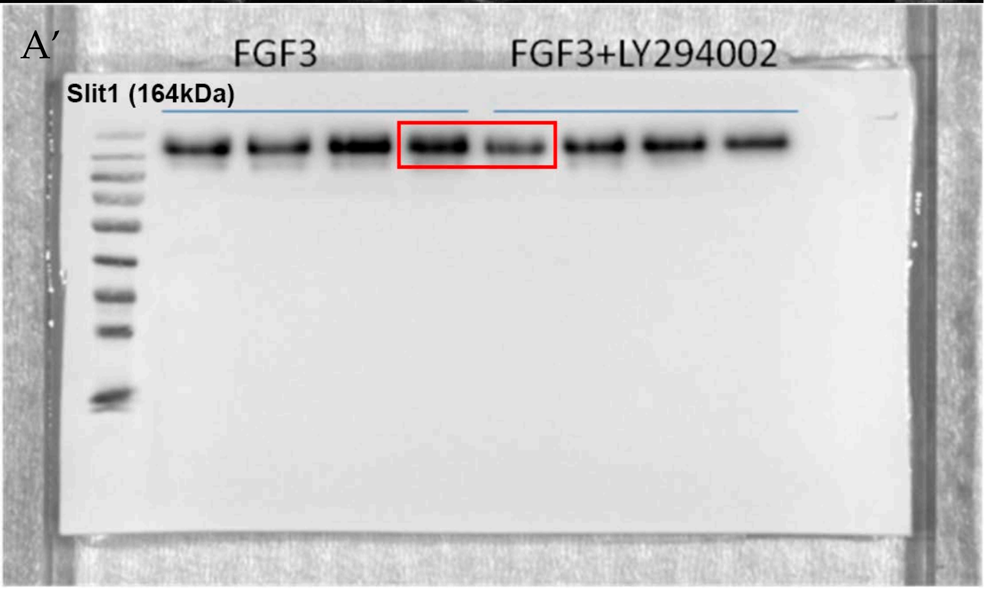
A′
FGF3
FGF3+LY294002
Slit1 (164kDa)

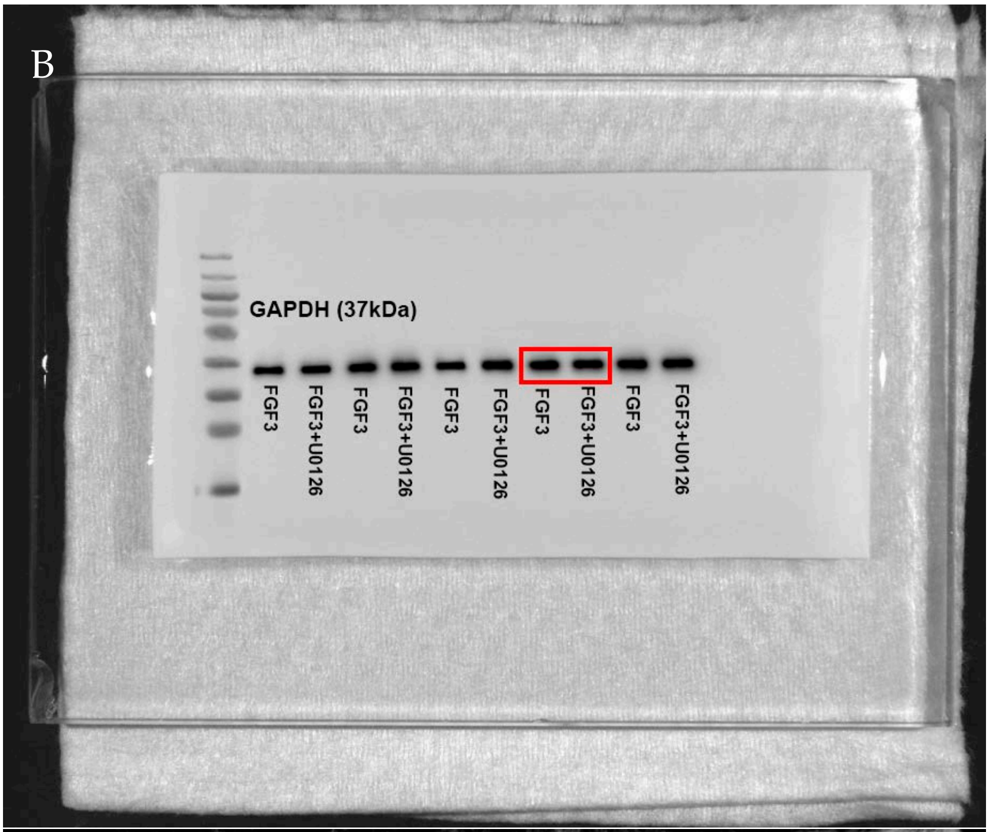
B
GAPDH (37kDa)
FGF3
FGF3+U0126
FGF3
FGF3+U0126
FGF3
FGF3+U0126
FGF3
FGF3+U0126
FGF3
FGF3+U0126

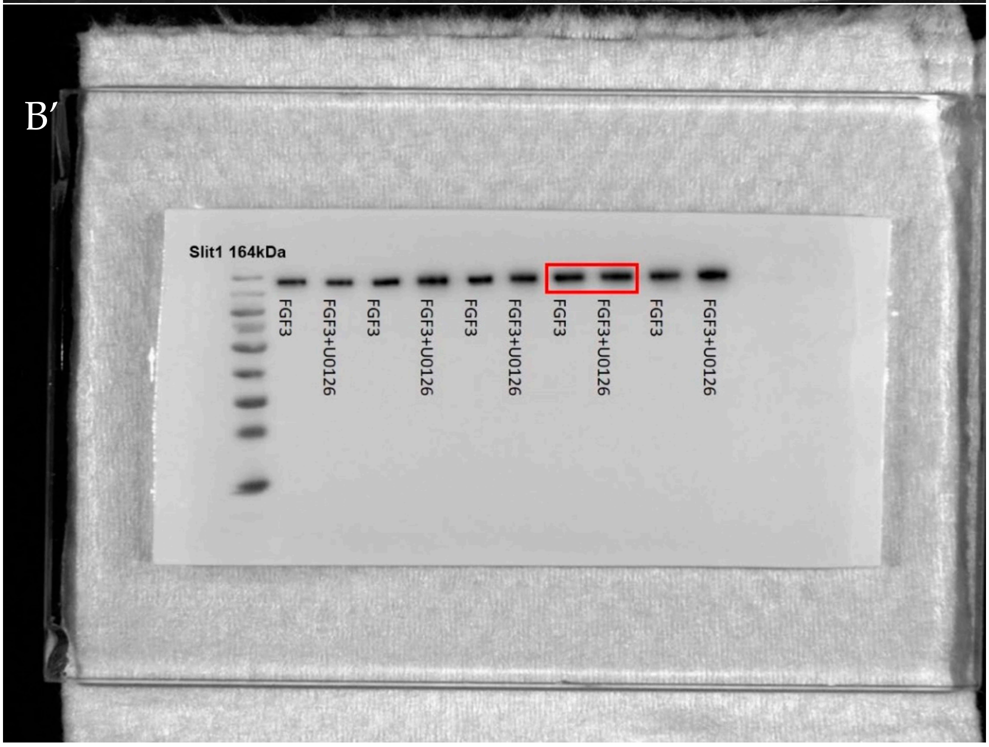
B′
Slit1 164kDa
FGF3
FGF3+U0126
FGF3
FGF3+U0126
FGF3
FGF3+U0126
FGF3
FGF3+U0126
FGF3
FGF3+U0126

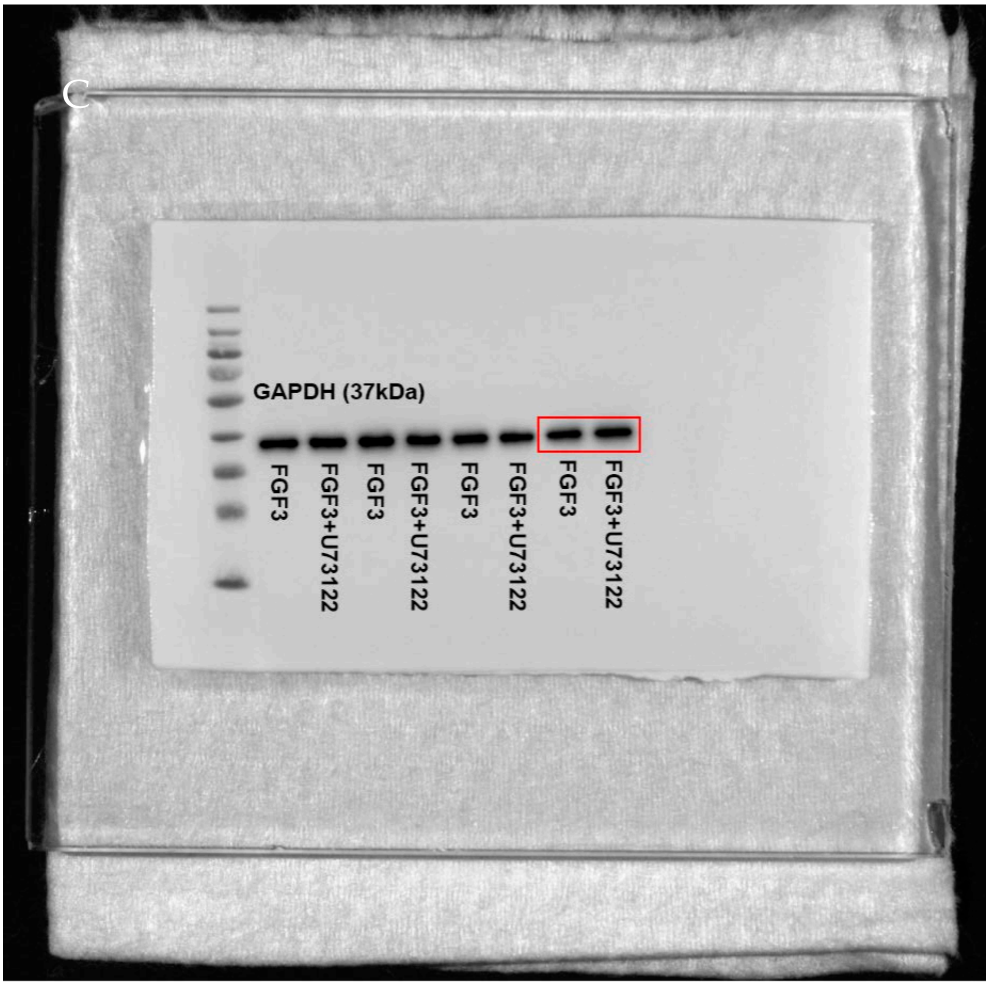
C
GAPDH (37kDa)
FGF3
FGF3+U73122
FGF3
FGF3+U73122
FGF3
FGF3+U73122
FGF3
FGF3+U73122

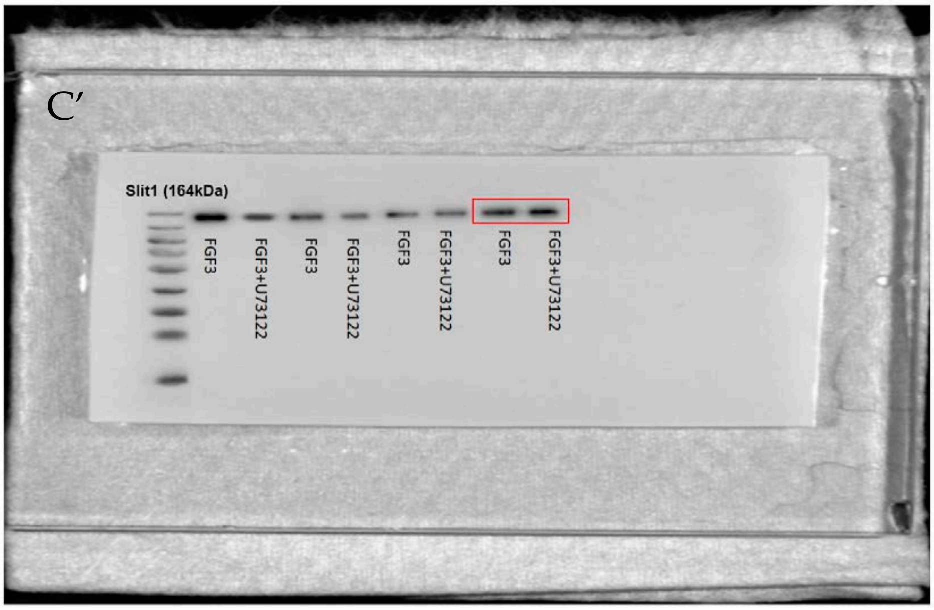
C′
Slit1 (164kDa)
FGF3
FGF3+U73122
FGF3
FGF3+U73122
FGF3
FGF3+U73122
FGF3
FGF3+U73122

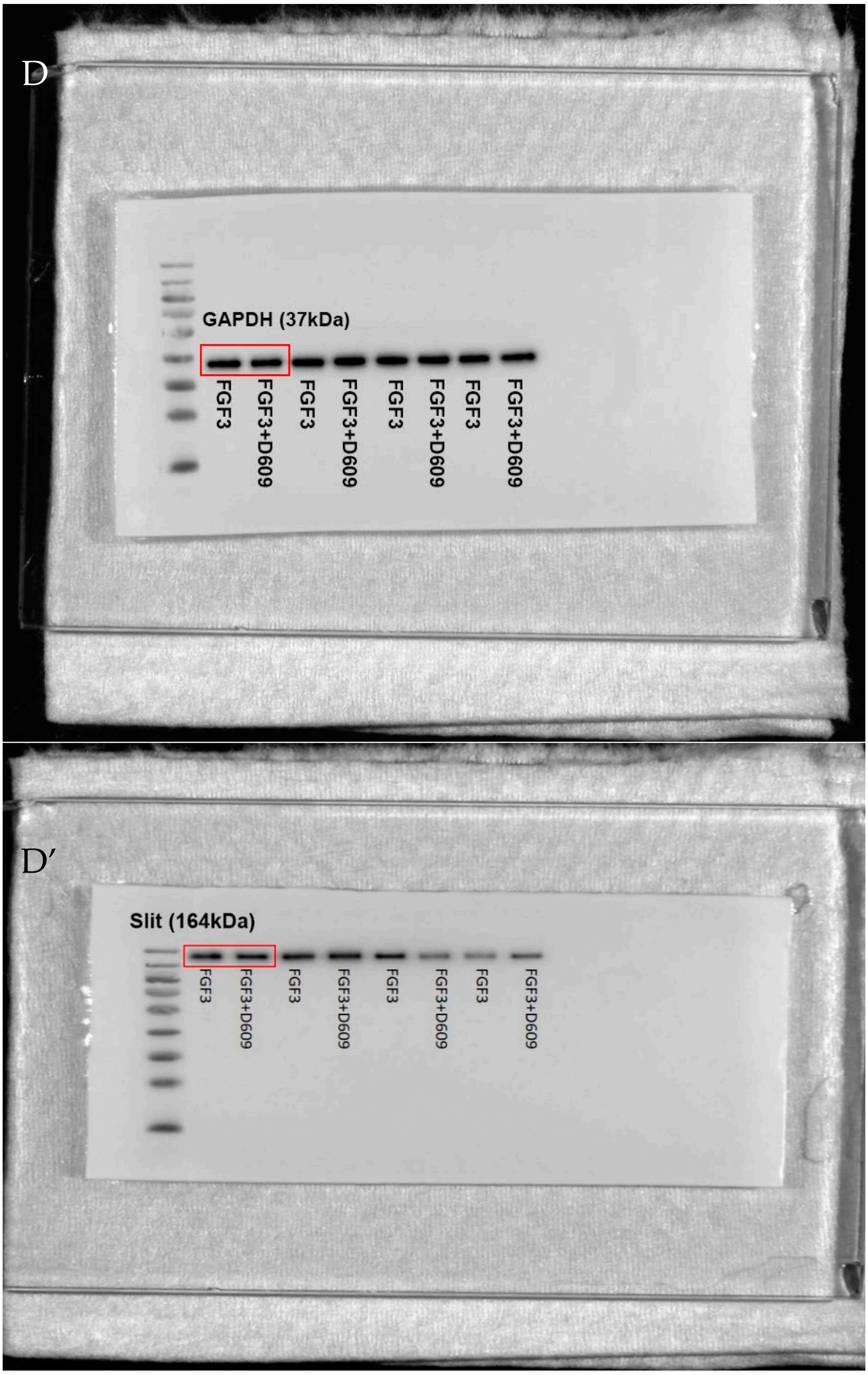

Figure S2. Western blot analysis of Slit1 expression regulated by FGF3 signaling via different pathways. **(A-D′)** GAPDH (37 kDa) serves as a loading control **(A,B,C,D).** Slit1 (164 kDa) expression in cells treated with FGF3 alone or FGF3 combined with LY294002 (a PI3K inhibitor) **(A′)**; U0126 (a MAPK/ERK inhibitor) **(B′)**; U73122 (a PLCγ inhibitor) **(C′)**; D609 (a PC-PLC inhibitor) **(D′)**. Red frame: representative wetern blot analysis selected in Figure 6.

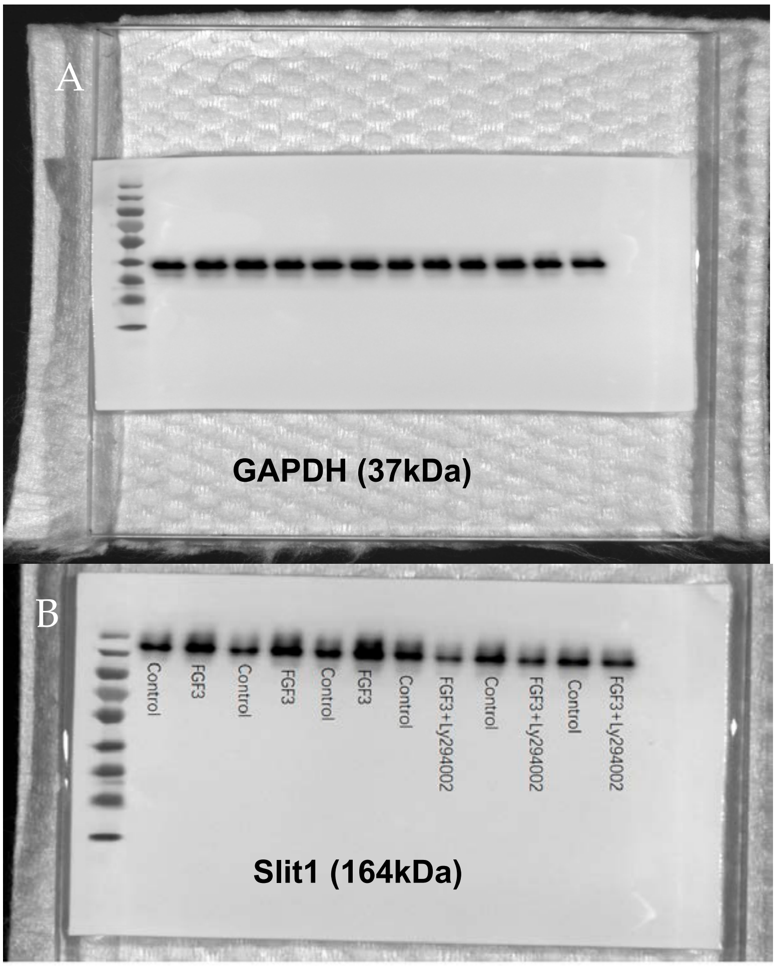

Figure S3. Western blot analysis of Slit1 expression of the thalamic explant. **(A)** GAPDH (37 kDa) serves as a loading control. **(B)** Slit1 (164 kDa) expression in cells separated inton two groups: FGF3 treatment group compared with control group, and FGF3+LY294002 treatment group compared with control group.
